# Stressed neuronal cells can recover from profound membrane blebbing, nuclear condensation and mitochondrial fragmentation, but not from cytochrome c release

**DOI:** 10.1038/s41598-023-38210-w

**Published:** 2023-07-08

**Authors:** Wenting You, Tao Zhou, Kèvin Knoops, Tos T. J. M. Berendschot, Marc A. M. J. van Zandvoort, Wilfred T. V. Germeraad, Birke Benedikter, Carroll A. B. Webers, Chris P. M. Reutelingsperger, Theo G. M. F. Gorgels

**Affiliations:** 1 University Eye Clinic Maastricht UMC+, Maastricht University Medical Center+, 6229 HX Maastricht, The Netherlands; 2 Department of Biochemistry, CARIM School for Cardiovascular Disease, Maastricht University, 6229 ER Maastricht, The Netherlands; 3 Department of Mental Health and Neuroscience, Maastricht University, 6229 ER Maastricht, The Netherlands; 4 The Maastricht Multimodal Molecular Imaging Institute, Maastricht University, 6229 ER Maastricht, The Netherlands; 5 Department of Molecular Cell Biology, CARIM School for Cardiovascular Disease, Maastricht University, 6229 ER Maastricht, The Netherlands; 6 Division of Hematology, Department of Internal Medicine, Maastricht University Medical Center, Maastricht, The Netherlands; 7 Institute of Molecular Cardiovascular Research, Universitätsklinikum Aachen, 52074 Aachen, Germany

**Keywords:** Cell death, Cellular neuroscience

## Abstract

Loss of neurons in chronic neurodegenerative diseases may occur over a period of many years. Once initiated, neuronal cell death is accompanied by distinct phenotypic changes including cell shrinkage, neurite retraction, mitochondrial fragmentation, nuclear condensation, membrane blebbing and phosphatidylserine (PS) exposure at the plasma membrane. It is still poorly understood which events mark the point of no return for dying neurons. Here we analyzed the neuronal cell line SH-SY5Y expressing cytochrome C (Cyto.C)-GFP. Cells were exposed temporarily to ethanol (EtOH) and tracked longitudinally in time by light and fluorescent microscopy. Exposure to EtOH induced elevation of intracellular Ca^2+^ and reactive oxygen species, cell shrinkage, neurite retraction, mitochondrial fragmentation, nuclear condensation, membrane blebbing, PS exposure and Cyto.C release into the cytosol. Removing EtOH at predetermined time points revealed that all phenomena except Cyto.C release occurred in a phase of neuronal cell death in which full recovery to a neurite-bearing cell was still possible. Our findings underscore a strategy of treating chronic neurodegenerative diseases by removing stressors from neurons and harnessing intracellular targets that delay or prevent trespassing the point of no return.

## Introduction

Apoptosis is a well-established form of regulated cell death, characterized by typical morphological and biochemical hallmarks, including cell shrinkage, membrane blebbing, mitochondrial fragmentation, chromatin condensation, caspase activation, and cleavage of poly(ADP)-ribose polymerase (PARP)1^1,2^. The sequence of events in the apoptotic process and the molecular mechanism of its induction and regulation have been studied for decades. Many investigators tried to find the “commitment point” of cell death – a molecular event marking the point of no return^3,4^.

Mitochondria play a pivotal role in activating caspase proteases through a pathway termed the intrinsic or mitochondrial pathway of apoptosis. An important event in the regulation of caspase activation and cell death is the process called mitochondrial outer membrane permeabilization (MOMP)^5^. Following MOMP, proteins including cytochrome c, Smac/DIABLO, endonuclease G (EndoG), and apoptosis inducing factor (AIF) are released from the mitochondrial intermembrane space (IMS) into the cytosol where they drive robust caspase activity and rapid apoptotic cell death^6^. Among the proteins released from mitochondria, cytochrome c is the most important and essential for mitochondrial dependent caspase activation^7^. Indeed, cells that lack cytochrome c failed to activate caspases following MOMP^8–10^. However, even in the absence of caspase activation, cells that undergo MOMP cannot survive. This is due to mitochondrial dysfunction, energy depletion, generation of free radicals, or the release of mitochondrial proteins that are toxic to cells independent of caspase activation^11,12^. This process is termed MOMP-dependent, caspase-independent cell death (CICD)^13^. Taken together, MOMP is generally considered a point of no return during apoptosis^3,4,13^.

Studies have reported that cells can recover from early stages in the cell death program^14–19^. Our previous study showed that neuronal PC12 cells could recover from various injuries if the cells had not yet experienced MOMP^20^. Interestingly, recent studies reported that cells could also recover from late stage of apoptosis, after cells passed important checkpoints that typically mark the point of no return, such as cytochrome c release from mitochondria, caspase activation, nuclear fragmentation, and the formation of apoptotic bodies^21–25^. This phenomenon is named “anastasis”, which means “rising to life” in Greek, to describe the reversal of apoptosis from the brink of cell death^26^. Anastasis has been observed in multiple types of cells both in vitro and in vivo^22,27^. Removal of the cell death stimulus was sufficient to allow cells to recover, suggesting that anastasis is an intrinsic capacity of cells. Studies have been performed to explore the key molecular mechanisms underlying anastasis^24,25,28,29^. The potential reversibility of execution stage apoptosis opened up new, previously unexpected therapeutic opportunities in the field of programmed cell death.

In the present study, we explored whether the reversibility of the cell death program is a general phenomenon for neurons, by testing various neuronal cell lines including differentiated SH-SY5Y cells, neuronal PC12 cells, HT22, and Neuro-2a cells, which are widely used in research of neurodegenerative diseases^30,31^. We studied the reversibility of multiple cell death events that are associated with loss of viability, including cell shrinkage, mitochondrial fragmentation, nuclear condensation, phosphatidylserine (PS) exposure, elevation of intracellular Ca^2+^, and ROS generation. Furthermore, employing mitochondrial cytochrome c release as an indicator, we studied whether neurons could recover from MOMP and thus show anastasis.

## Results

### EtOH, Glutamate and MPP + induced cell death in differentiated SH-SY5Y cells

**Figure 1:**
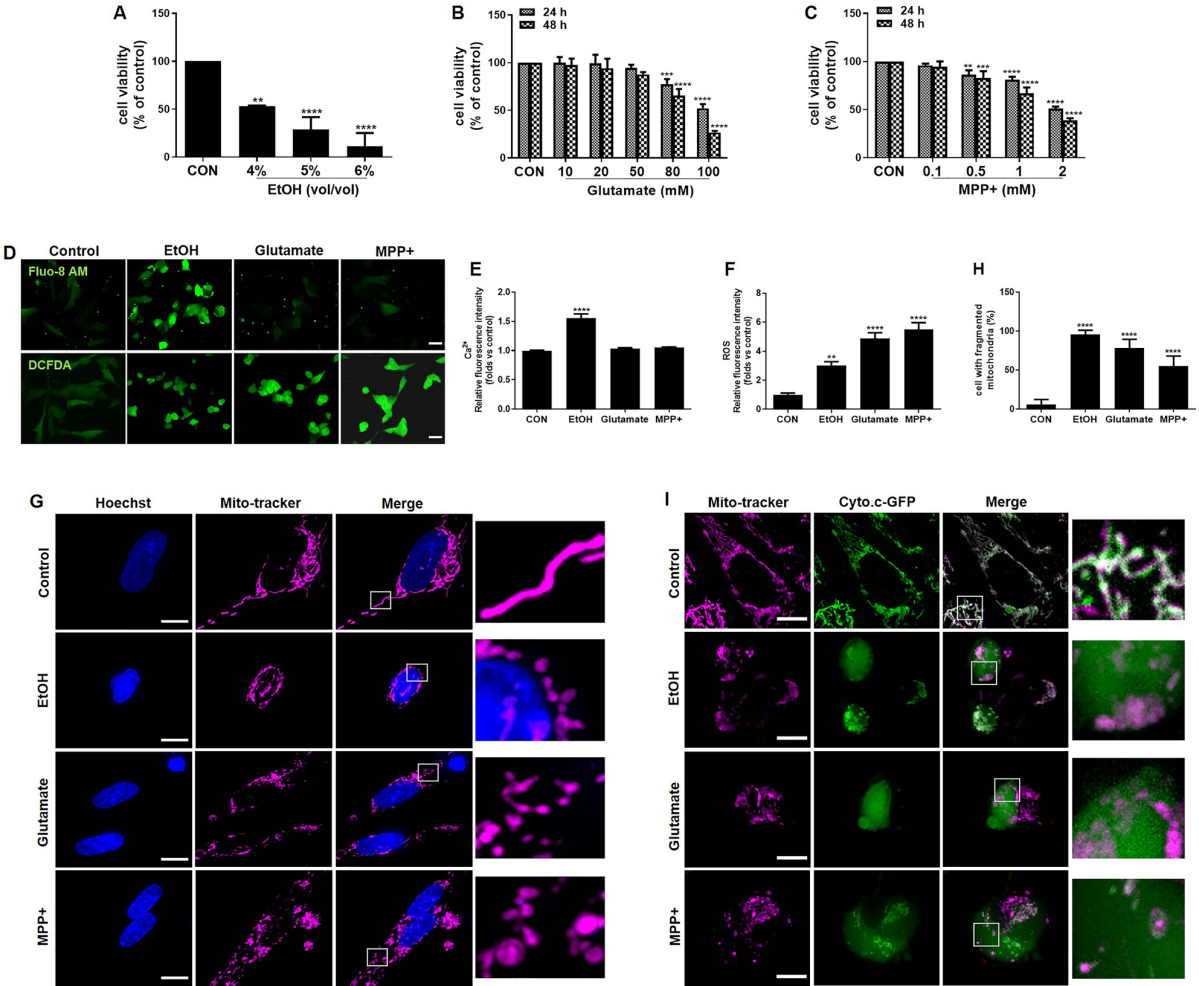
EtOH, Glutamate, MPP + induced cell death in differentiated SH-SY5Y cells. Differentiated SH-SY5Y cells were treated with EtOH (4%, 5%, 6%; vol/vol) for 24 h (**A**); Glutamate (10 mM, 20 mM, 50 mM, 80 mM, 100 mM) for 24 h or 48 h (**B**); MPP + (0.1 mM, 0.5 mM, 1 mM, 2 mM) for 24 h or 48 h (**C**). PrestoBlue™ assay was performed to assess cell viability expressed as percentage of control group (100%). (**D**) Intracellular Ca^2+^ and ROS levels were measured by Fluo-8 AM and DCFDA staining, respectively, after EtOH (5%, vol/vol; 3 h), Glutamate (100 mM; 24 h), and MPP + (2 mM; 24 h) treatment. Scale bar: 20 μm. (**E**) and (**F**) Quantitative analysis in terms of relative fluorescence intensity of Ca^2+^ and ROS. EtOH (5%, 3 h), Glutamate (100 mM, 24 h) and MPP + (2 mM, 24 h) induced mitochondrial fragmentation in differentiated SH-SY5Y cells as shown in confocal images (**G**) and in quantification (**H**). Mitochondria and nuclei were visualized by staining with Mito-tracker (magenta) and Hoechst33342 (blue). Scale bar: 10 μm. (**I**) Cyto.c-GFP (green) expressing differentiated SH-SY5Y cells were stained with Mito-tracker (magenta). Cyto.c-GFP colocalizes with mitochondria in the control group and translocates to the cytoplasm after EtOH (8 h), Glutamate (24 h), and MPP + (24 h) treatment. Scale bar: 10 μm. Data are presented as the mean ± SEM based on three independent experiments. **p < 0.01; ****p < 0.0001, versus control group. CON: control. EtOH: ethanol.

We first examined the dose effect of EtOH, Glutamate, and MPP + on cell viability in differentiated SH-SY5Y cells. Cells were treated with different concentrations of EtOH (4%, 5%, or 6%) for 24 h, Glutamate (10, 20, 50, 80, or 100 mM) for 24 or 48 h, MPP + (0.1, 0.5, 1, or 2 mM) for 24 or 48 h. Subsequently, cell viability was determined by the PrestoBlue assay. EtOH, Glutamate, and MPP + all produced a concentration- and time-dependent decrease in cell viability (Fig. 1A,B,C). EtOH (5%, vol/vol), Glutamate (100 mM) and MPP + (2 mM), which induced around 50% cell death after 24 h treatment, were chosen as working concentrations in all following experiments. To investigate the cellular mechanism behind the cell death response to EtOH, Glutamate, and MPP + treatment, we performed a series of experiments to evaluate the intrinsic mitochondrial pathway of apoptosis. Intracellular Ca^2+^ level was examined using the Fluo-8 AM fluorescence dye. Fluorescence intensity increased significantly when cells were treated with EtOH, but no significant changes were observed after treatment with Glutamate and MPP + (Fig. 1D,E). Intracellular ROS level was examined using the DCFDA fluorescence dye. Fluorescence intensity increased significantly after treatment with EtOH, Glutamate, and MPP + (Fig. 1D,F). We stained cells with Mito-tracker to show mitochondrial structure changes; the results showed that EtOH, Glutamate, and MPP + treatment all induced significant fragmentation of the mitochondrial network into small individual mitochondria (Fig. 1G,H). Next, we transfected cells with a cytochrome c-GFP (Cyto.c-GFP) fusion construct that has been previously shown to accumulate in the mitochondrial intermembrane space and to be released into the cytosol during MOMP^32^. Following transfection, we observed that Cyto.c-GFP co-localized with mitochondria and appeared as a thin filamentous network in the control cells. When cells were treated with EtOH, Glutamate, and MPP + , we observed the release of Cyto.c-GFP into the cytosol indicated by an evenly distributed signal throughout the cytosol, while the Mito-tracker image showed distinct, fragmented mitochondria (Fig. 1I).

### Recovery of differentiated SH-SY5Y cells from EtOH induced cell death program

**Figure 2:**
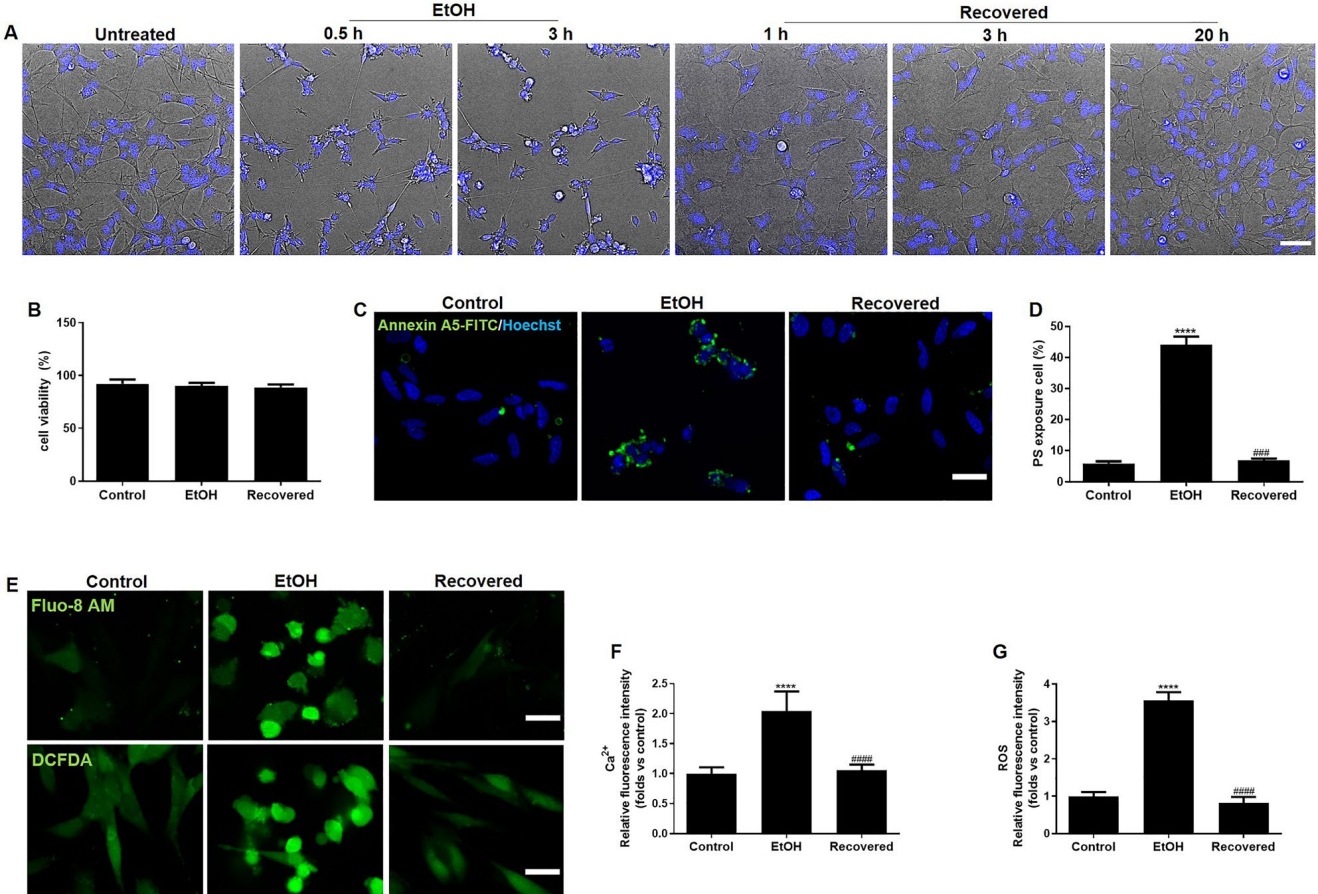
Recovery of differentiated SH-SY5Y cells from EtOH induced cell death. (**A**) Live cell imaging of differentiated SH-SY5Y cells before, during, and after exposure to EtOH. The same cells before EtOH treatment (Untreated), treated with 5% EtOH in culture medium for 0.5 h and 3 h, and then washed away EtOH and further cultured with fresh medium (Washed). Nuclei were visualized by staining with Hoechst33342. 15 positions were chosen randomly for imaging. Scale bar: 20 μm. (**B**) Cell viability of differentiated SH-SY5Y cells before (control), during (EtOH, 3 h), and after removing EtOH and further culturing in fresh culture medium for another 20 h (Washed). (**C**) Reversible phosphatidylserine (PS) externalization in EtOH treated differentiated SH-SY5Y cells and quantification data (**D**). Annexin A5-FITC labeled exposed PS (green), nuclei (blue). Scale bar: 20 μm. (**E**) Reversible elevation of intracellular Ca^2+^ and ROS in EtOH treated differentiated SH-SY5Y cells shown by Fluo-8 AM and DCFDA staining, respectively, and quantification data (**F**–**G**). Scale bar: 20 μm. Data are presented as the mean ± SEM based on three independent experiments. ****p < 0.0001, versus control group; ^####^*p* < 0.0001, versus EtOH group. EtOH: ethanol.

To test whether the cell death program is a reversible process, we exposed differentiated SH-SY5Y cells to EtOH (5%, vol/vol) until they showed typical hallmarks of apoptosis. We then washed away EtOH and cultured the cells further in fresh medium to allow the cells to show recovery. Live cell imaging results showed that after exposure to EtOH for 3 h, cells displayed morphological hallmarks of apoptosis, including cell shrinkage, plasma membrane blebbing, and nuclear condensation (Fig. 2A). Upon the removal of EtOH by washing, the same cells that had exhibited these morphological hallmarks of apoptosis, recovered to normal morphology (Fig. 2A). Cell viability results showed that during this recovery process, there was no significant cell death (Fig. 2B). Exposure of phosphatidylserine (PS) on the outer plasma membrane has been established as an early marker of neuronal apoptosis^33^. Using annexin A5-FITC, we observed that around 40% of the differentiated SH-SY5Y cells exposed PS at the plasma membrane of the cell body after 3 h of exposure to EtOH. Interestingly, removal of EtOH by washing reversed PS exposure back to base levels within 15 h (Fig. 2C,D). Next, we measured the intracellular Ca^2+^ and ROS levels, using Fluo-8 AM and DCFDA, respectively. EtOH caused an elevation of these levels in differentiated SH-SY5Y cells, but, after removing EtOH for 15 h, the levels returned to normal (Fig. 2E,F,G). Together, these results suggest that neuronal cells can recover from early stages of apoptosis when the cell death trigger is removed.

### SH-SY5Y cells can recover from mitochondrial fragmentation

**Figure 3:**
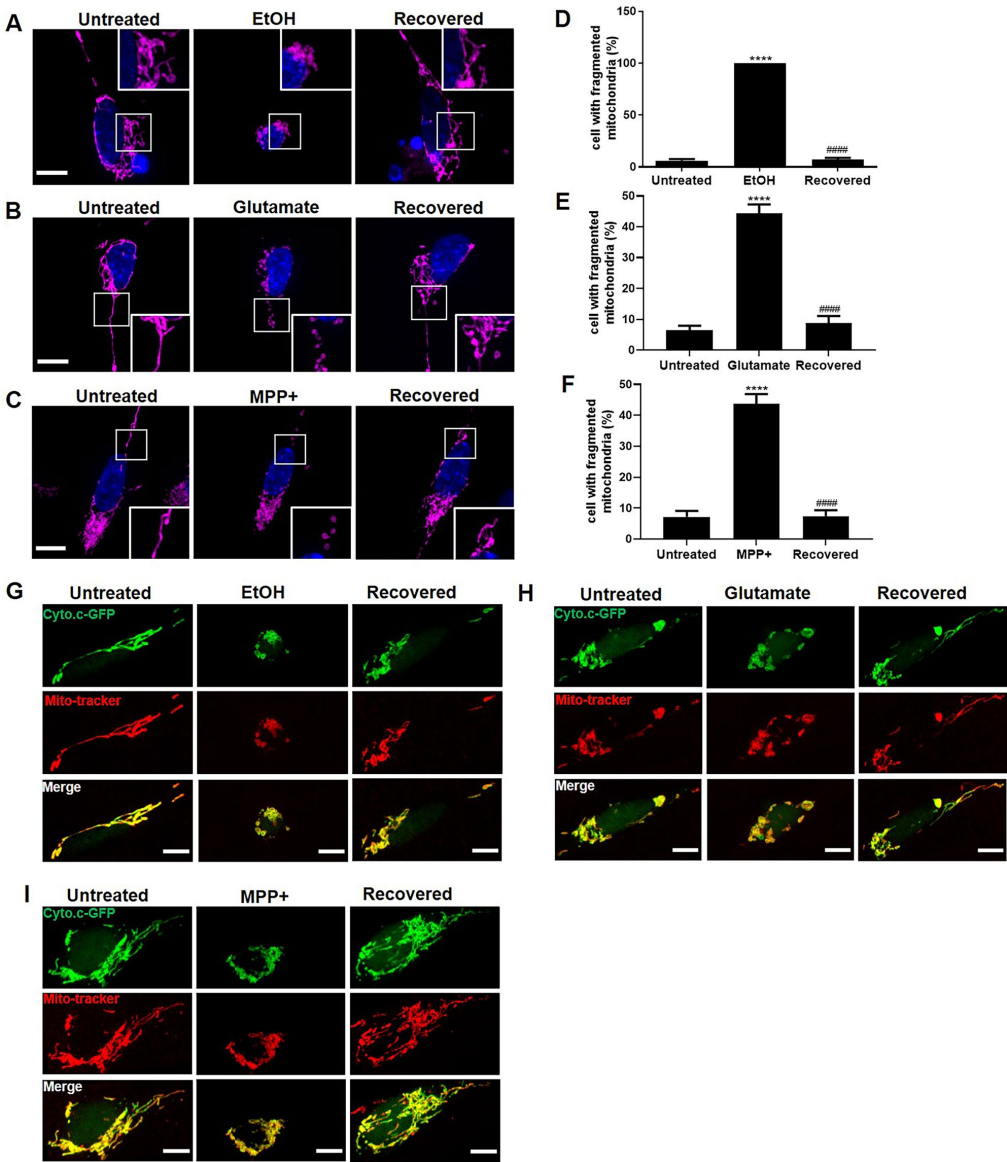
Reversible mitochondrial fragmentation in EtOH, Glutamate, and MPP + treated differentiated SH-SY5Y cells. (**A**–**C**) Same differentiated SH-SY5Y cells were monitored to study the reversibility of mitochondrial fragmentation before, during, and after exposure to EtOH (5%, vol/vol; 2 h), Glutamate (100 mM; 10 h), and MPP + (2 mM; 10 h) by live cell imaging. Mitochondria were visualized by staining with Mito-tracker (magenta) and nuclei by Hoechst33342 (blue). Scale bar: 10 μm. (**D**–**F**) Quantification result of cells with fragmented mitochondria. (**G**–**I**) Differentiated SH-SY5Y cells expressing Cyto.c-GFP (green) were stained with Mito-tracker (red) and treated with EtOH (5%, vol/vol), Glutamate (100 mM), and MPP + (2 mM). Same cells were monitored to observe the dynamics of mitochondria and the distribution of cytochrome c before, during, and after EtOH, Glutamate, or MPP + treatment by live cell imaging. Scale bar: 10 μm. Data are presented as the mean ± SEM based on three independent experiments. ****p < 0.0001, *vs.* control group; ^####^*p* < 0.0001, versus EtOH, Glutamate, or MPP + group. EtOH: ethanol.

In order to observe directly the reversibility of mitochondrial fragmentation, we used confocal live cell imaging to monitor the dynamics of mitochondria in individual SH-SY5Y cells. As shown in Fig. 3A,B,C, mitochondria normally appeared as a filamentous network in untreated cells. After exposure to EtOH, Glutamate, or MPP + , the shape of mitochondria changed from filamentous to spherical. Interestingly, after removing the cell death stimulus by washing and further culturing cells in fresh medium, fragmented mitochondria regained their normal morphology. Quantification showed that, almost 100% of the cells had mitochondrial fragmentation after 2 h exposure to EtOH, while more than 90% of the cells regained normal mitochondrial structure after removing EtOH (Fig. 3D). In Glutamate and MPP + treated SH-SY5Y cells for 10 h, around 40% of cells had fragmented mitochondria. By monitoring the same cells after washing away Glutamate and MPP + , we observed that most of the cells with fragmented mitochondria regained normal mitochondrial structure (Fig. 3E,F). In order to study whether cytochrome c had been released from mitochondria during this stage of reversible mitochondrial fragmentation, we used Cyto.c-GFP transfected SH-S5Y cells to monitor the translocation of cytochrome c into the cytosol. Live imaging of Mito-tracker labeled Cyto.c-GFP SH-S5Y cells showed that Cyto.c-GFP and Mito-tracker co-localized as a filamentous network in untreated SH-SY5Y cells. EtOH treatment induced fragmentation of the Mito-tracker labeled filamentous structures into spherical structures. During the fragmentation, Cyto.c-GFP remained co-localized with Mito-tracker indicating that cytochrome c had not been released from mitochondria. Removal of EtOH caused a reorganization of mitochondria into filamentous structures with retained co-localization of Mito-tracker and Cyto.c-GFP (Fig. 3G). Similar results were observed in Glutamate and MPP + treated SH-SY5Y cells (Fig. 3H,I).

### Reversible mitochondrial fragmentation in various neuronal cell lines

**Figure 4:**
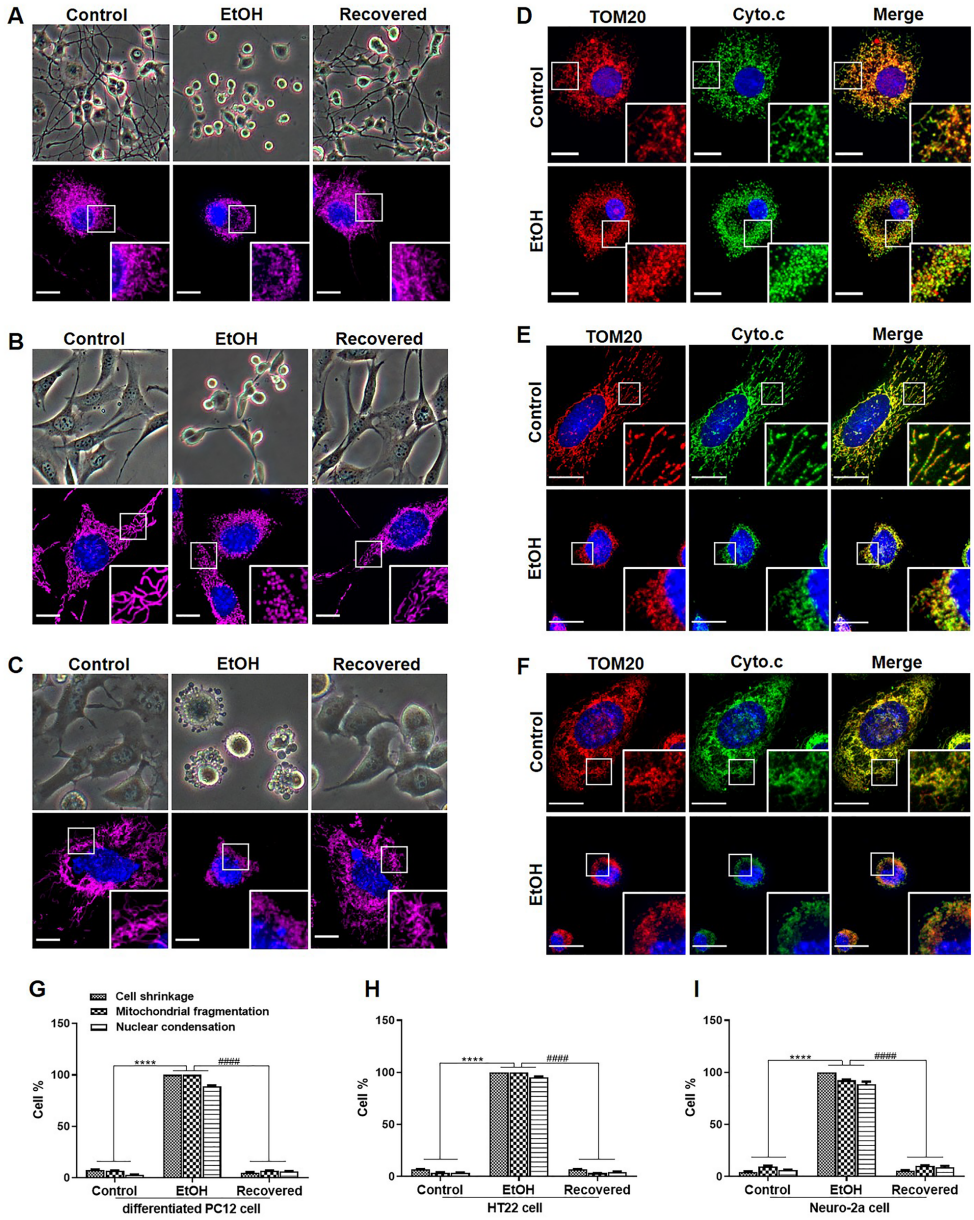
Reversible mitochondrial fragmentation in EtOH treated differentiated PC12 cells, HT22 cells, and Neuro-2a cells. Live cell imaging of differentiated PC12 cells (**A**), HT22 cells (**B**), and Neuro-2a cells (**C**) before EtOH (5%, vol/vol) treatment (Control), after EtOH treatment (EtOH, 2 h), and after removing EtOH and allowing cells to recover in fresh culture medium for 20 h (Recovered). Mitochondria were stained by Mito-tracker (magenta) and nuclei by Hoechst33342 (blue). Scale bar: 10 μm. Confocal microscope images of representative control and EtOH treated differentiated PC12 cells (**D**), HT22 cells (**E**), and Neuro-2a cells (**F**) stained with cytochrome c (Cyto.c, green), TOM20 (red) antibodies and DAPI (blue). Scale bar: 10 μm. (**G**–**I**) Quantification results of control, EtOH treated, and recovered differentiated PC12 cells, HT22 cells, and Neuro-2a cells that displayed cell shrinkage, mitochondrial fragmentation, and nuclear condensation. Data are presented as the mean ± SEM based on three independent experiments. ****p < 0.0001, *vs.* control group; ^####^*p* < 0.0001, versus EtOH group. EtOH: ethanol.

The recovery of SH-SY5Y cells, observed before mitochondrial cytochrome c release prompted us to investigate whether this was a general phenomenon by studying various neuronal cell lines. EtOH exposure was applied to differentiated PC12 cells, HT22 cells, and Neuro-2a cells. As shown in Fig. 4A, 2 h EtOH treatment induced significant neurite retraction, mitochondrial fragmentation, and nuclear condensation in differentiated PC12 cells. Interestingly, cells regrew new neurites, and regained normal mitochondrial and nuclear morphology after removal of EtOH as reported previously with time-lapse video recording^20^. Similarly, 2 h EtOH treatment caused cell shrinkage, membrane blebbing, mitochondrial fragmentation, and nuclear condensation in HT22 and Neuro-2a cells. These cell types also recovered morphologically within 20 h after removal of EtOH (Fig. 4B,C). Quantification showed that more than 90% of cells that responded morphologically to EtOH recovered to normal morphology after EtOH removal (Fig. 4G,H,I). Also, mitochondrial fragmentation was not accompanied by any detectable release of cytochrome c into the cytosol in these three cell lines as revealed by anti-cytochrome c and anti-TOM20 antibodies (Fig. 4D,E,F).

### SH-SY5Y cells did not recover from cytochrome c release

**Figure 5:**
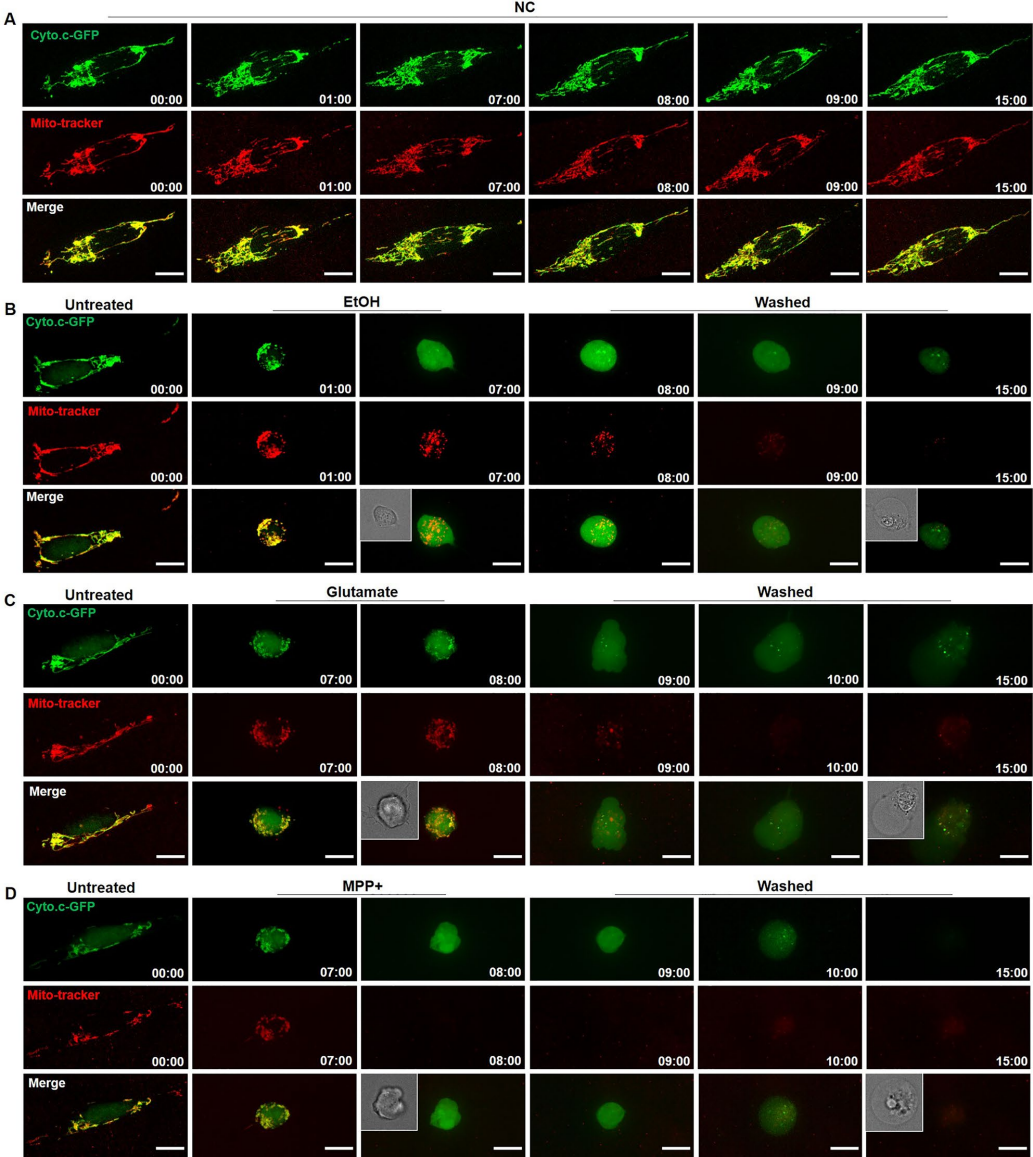
Non-reversible cytochrome c release in EtOH, Glutamate, and MPP + treated differentiated SH-SY5Y cells. (**A**) SH-SY5Y cells expressing Cyto.c-GFP (green) were monitored by real-time live cell imaging without any treatment as negative control (NC) group. Mitochondria were stained by Mito-tracker (magenta). Scale bar: 10 μm. (**B**–**D**) SH-SY5Y cells expressing Cyto.c-GFP (green) were monitored by real-time live cell imaging before, during, and after EtOH (5%, vol/vol), Glutamate (100 mM), and MPP + (2 mM) treatment for indicated time. Mitochondria were stained by Mito-tracker (magenta). Scale bar: 10 μm. 30–50 cells were imaged in each group.

Next, we investigated whether cells can also recover after cytochrome c had been released from mitochondria. As shown in Fig. 5A, untreated SH-SY5Y cells expressing Cyto.c-GFP, were stained with Mito-tracker and imaged to verify that imaging and staining procedures were not cytotoxic. In Fig. 5B,C,D, EtOH, Glutamate, and MPP + were applied to SH-SY5Y cells expressing Cyto.c-GFP until cytochrome c was released from mitochondria and Cyto.c-GFP in the cells became uniformly distributed over the entire cytosol. Then, the cell death stimuli were removed by washing, and cells were further cultured in fresh medium. By monitoring individual cells by live cell imaging after removing the cell death stimuli, we did not observe any cells that recovered from cytochrome c release (Fig. 5B,C,D).

### Study of reversibility of the cell death program in STS and EtOH treated HeLa cells

**Figure 6:**
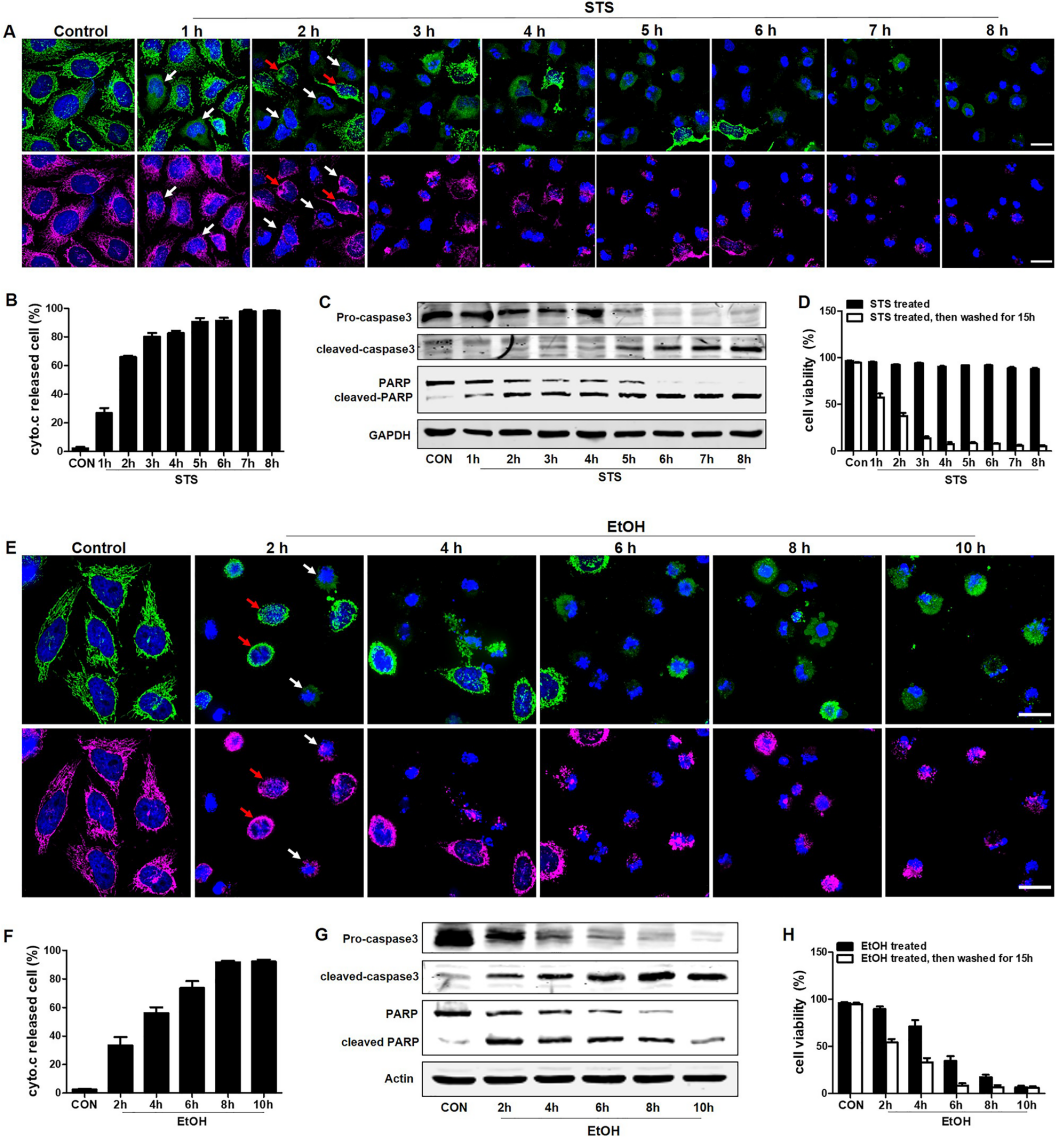
EtOH and staurosporine (STS) induced apoptosis in HeLa cells, and cell survival in cell population after removal of EtOH and STS. (**A**) Confocal images of fixed HeLa cells after treatment with STS (250 nM) for (1, 2, 3, 4, 5, 6, 7, 8) h. Mitochondria were stained by TOM20 antibody (magenta), cytochrome c by cyto.c antibody (green), and nuclei by DAPI (blue). White arrows indicate cells with cytochrome c released into the cytosol, and red arrows indicate cells with cytochrome c contained within the mitochondria. Scale bar: 20 μm. (**B**) Percentage of cytochrome c released HeLa cells treated with STS (250 nM) for the indicated time. Data are presented as the mean ± SEM based on three independent experiments. At least 300 cells were imaged for each experiment at each time point. (**C**) Western blot analysis of pro-caspase3, cleaved-caspase3, PARP, and cleaved-PARP in STS (250 nM) treated Hela cells. (**D**) Cell viability of HeLa cells after STS (250 nM) treatment for the indicated time or after STS treatment, then washing away STS and further culturing cells in fresh culture medium for another 15 h. This was measured by Hoechst33342 and PI double staining. (**E**) Confocal images of fixed Hela cells expressing Cyto.c-GFP after treatment with EtOH (4.3%, vol/vol) for 2, 4, 6, 8, 10 h. Mitochondria were stained by Mito-tracker (magenta), and nuclei by Hoechst33342 (blue). White arrows indicate cells with cytochrome c released into the cytosol, and red arrows indicate cells with cytochrome c contained within the mitochondria. Scale bar: 20 μm. (**F**) Percentage of cytochrome c releasing HeLa cells treated with EtOH (4.3%, vol/vol) for the indicated time. Data are presented as the mean ± SEM based on three independent experiments. At least 300 cells were measured for each experiment at each time point. (**G**) Western blot analysis of pro-caspase3, cleaved-caspase3, PARP, and cleaved-PARP in EtOH (4.3%, vol/vol) treated Hela cells. (**H**) Cell viability of HeLa cells after EtOH (4.3%, vol/vol) treatment for the indicated time or after EtOH treatment, then washing away EtOH and further culturing cells in fresh culture medium for another 15 h. CON: control. EtOH: ethanol.

Recent studies reported that HeLa cells that were treated with lethal doses of staurosporine (STS) and EtOH could recover from cytochrome c release, caspase activation, PARP activation, DNA damage, and even apoptotic bodies formation^22–24,26^. In order to analyze and understand why differentiated SH-SY5Y cells cannot recover from cytochrome c release, we studied STS (250 nM) and EtOH (4.3%, vol/vol) treated HeLa cells as a positive control for the recovery of cells from cytochrome c release. As shown in Fig. 6A, HeLa cells were fixed at different times following STS-induced apoptotic treatment. The distribution of cytochrome c was visualized by immunostaining using anti-cytochrome c antibody. The morphology of mitochondria was revealed by staining the cells with an antibody against TOM20, a mitochondrial outer membrane marker^34^. Cells in which cytochrome c was distributed in the cytosol, while their mitochondria were fragmented and spherical, were counted as cytochrome c released cells. The quantification result showed that the percentage of cells displaying cytochrome c release steadily increased during continuous STS treatment from 1 to 8 h (Fig. 6B). Western blotting results showed that STS increased the expression of cleaved-caspase 3 protein in a time-dependent manner, and the expression of pro-caspase 3 decreased simultaneously. STS also increased the cleavage of poly (ADP)-ribose polymerase-1 (PARP), which is a substrate of caspase 3, in a time-dependent manner (Fig. 6C). At the same time, cell viability measured by Hoechst33342/PI double staining showed that an increasing percentage of cells could not survive after removing STS when cells had been treated with STS for 1 to 8 h (Fig. 6D). Similarly, in EtOH treated HeLa cells, the percentage of cells displaying cytochrome c release increased in a time-dependent manner from 2 to 10 h (Fig. 6E,F). Protein expression of cleaved caspase 3 and PARP also increased in a time-dependent manner (Fig. 6G). Cell viability result showed that more and more cells did not survive after washing EtOH when cells had been treated with EtOH for 2 to 10 h (Fig. 6H). These results suggest that most HeLa cells did not survive and recover from cytochrome c release and caspase 3 activation after removing the cell death stimulus.

**Figure 7:**
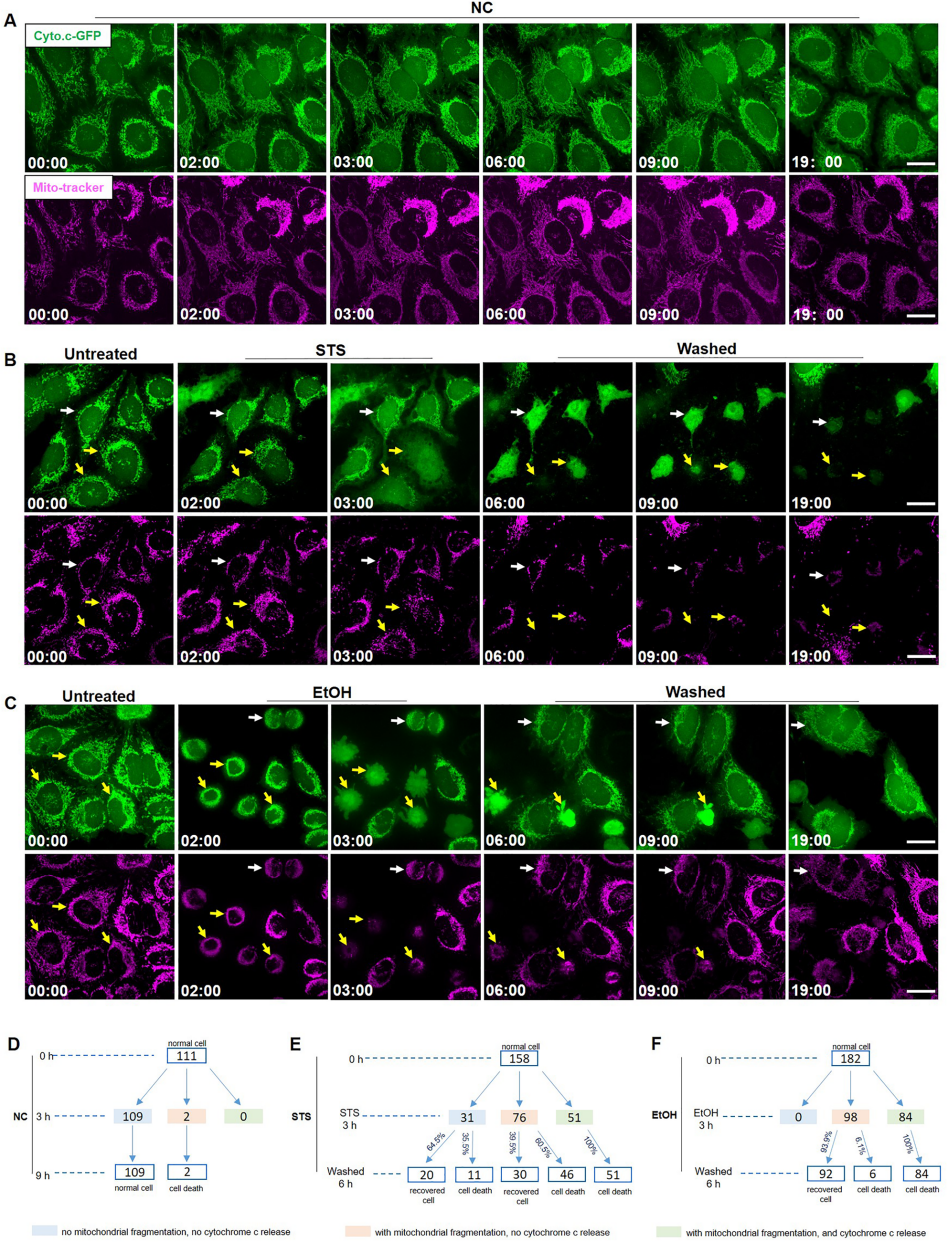
Study of recovery of HeLa cells from cytochrome c release induced by EtOH and STS. (**A**) HeLa cells expressing Cyto.c-GFP (green) were monitored by real-time live cell imaging without any treatment. Mitochondria were stained by Mito-tracker (magenta). 111 cells were imaged in negative control (NC) group. Scale bar: 20 μm. (**B**) HeLa cells expressing Cyto.c-GFP (green) were monitored by real-time live cell imaging before STS (250 nM) treatment, during STS treatment, and after washing away STS and further culturing cells in fresh culture medium for indicated time (Washed). Mitochondria were stained by Mito-tracker (magenta). 155 cells were imaged in STS group. Scale bar: 20 μm. (**C**) HeLa cells expressing Cyto.c-GFP (green) were monitored by real-time live cell imaging before EtOH (4.3%, vol/vol) treatment, during EtOH treatment, and after washing away EtOH and further culturing cells in fresh culture medium for indicated time (Washed). Mitochondria were stained by Mito-tracker (magenta). 180 cells were imaged in EtOH group. Scale bar: 20 μm. EtOH: ethanol. Time presented as hr:min. (**D**) Cell counting of HeLa cells expressing Cyto.c-GFP without treatment and imaged at 0, 3, 9 h. (**E** and **F**) cell counting and quantification of HeLa cells expressing Cyto.c-GFP before, during EtOH (4.3%, 3 h) or STS (250 nM, 3 h) treatment, or after treatment and washing EtOH and STS for another 6 h.

In order to see whether there were exceptions of HeLa cells that did recover after cytochrome c release, we monitored Hela cells expressing Cyto.c-GFP over time by real-time live cell imaging. As shown in Fig. 7A, HeLa cells in the negative control (NC) group, were stained with Mito-tracker and imaged to verify that imaging and staining procedures were not cytotoxic. In normal HeLa cells, before cytochrome c was released from mitochondria, the distribution patterns of both Cyto.c-GFP and Mito-tracker were the same and co-localized, appearing as a filamentous network. As shown in Fig. 7, we followed cells which displayed cytochrome c released from mitochondria into the cytosol after STS treatment for 3 h, and which failed to recover after removing STS (yellow arrows in Fig. 7B). In addition, we observed cells with cytochrome c localized mainly in the mitochondria, while their mitochondria had a fragmented structure, which also failed to recover after removing STS (white arrow in Fig. 7B). Similarly, in EtOH treated HeLa cells, we observed that cells with cytochrome c released from mitochondria into the cytosol after EtOH treatment for 3 h, failed to recover after removing EtOH (yellow arrows in Fig. 7C). In addition, cells with fragmented mitochondria and cytochrome c localized mainly in mitochondria recovered to normal morphology after removing the EtOH by washing (white arrows in Fig. 7C).

Next, we examined the distribution pattern of cytochrome c and the morphology of mitochondria in HeLa cells subjected to the STS and EtOH treatment for 3 h. We noticed that cells could be classified into three different categories: (1) cells with no mitochondrial fragmentation and no detectable release of cytochrome c; (2) cells with mitochondrial fragmentation and no cytochrome c release; (3) cells with mitochondrial fragmentation and with cytochrome c release. The statistical distribution of these three categories of cells and their recovery after removing the STS and EtOH is shown Fig. 6D,E,F. We did not observe any cells recovering from cytochrome c release after removing the cell death stimulus, either STS or EtOH.

A small difference in survival was noticed between the two cell death triggers. In EtOH treated HeLa cells, more than 90% of cells without cytochrome c release, could recover from mitochondrial fragmentation after washing EtOH. In STS treated Hela cells, there was, after washing STS, still 60.5% of cell death in cells with mitochondrial fragmentation but without cytochrome c release, and even 35.5% of cell death in cells with normal mitochondrial structure and cytochrome c distribution.

### Effects of caspase inhibitor Z-VAD-FMK on cell shrinkage and membrane blebbing

**Figure 8:**
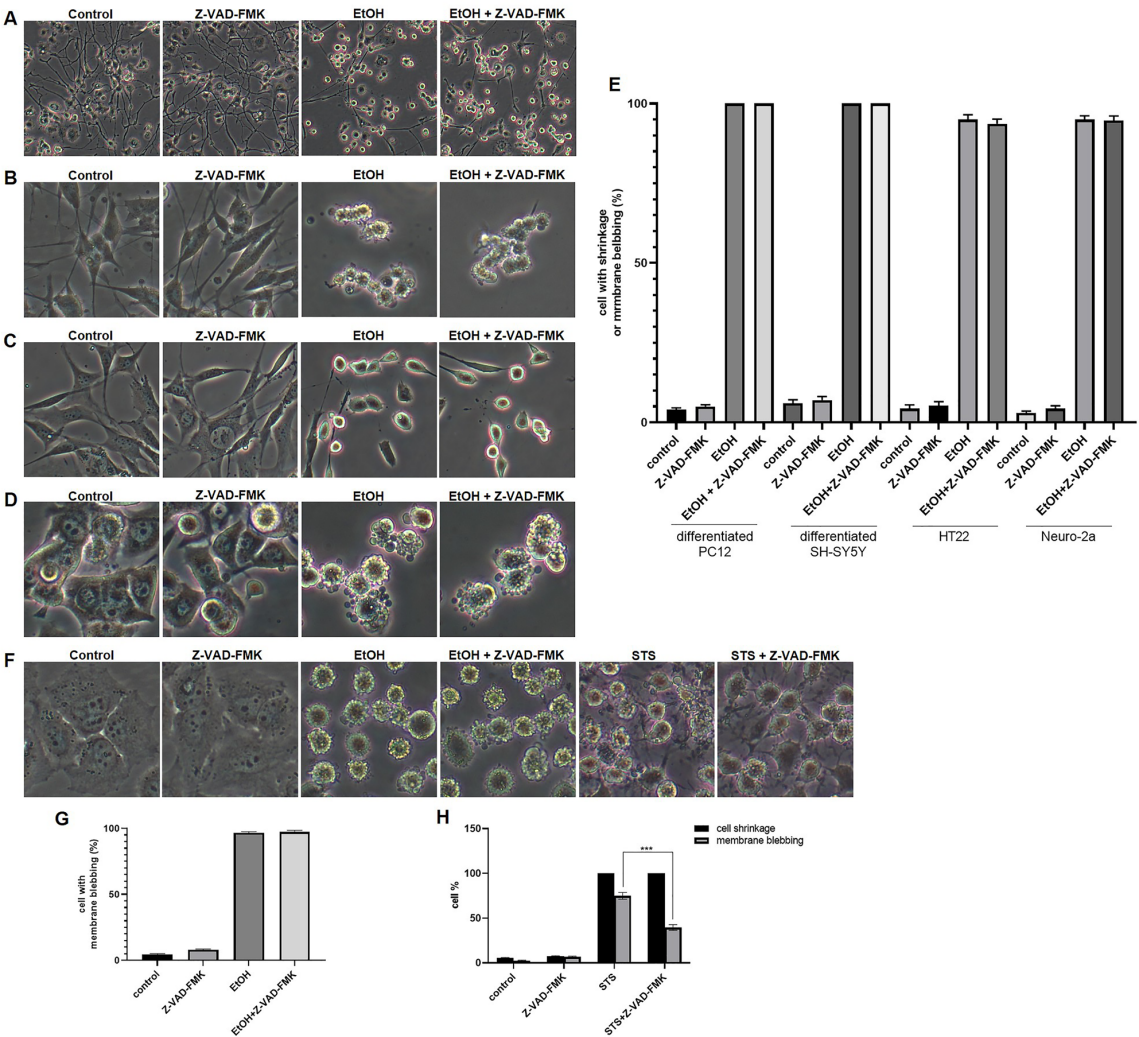
Effect of caspase inhibitor Z-VAD-FMK on cell shrinkage and membrane blebbing. Bright field images of differentiated PC12 cells (**A**), differentiated SH-SY5Y cells (**B**), HT22 cells (**C**), and Neuro-2a cells (**D**) without treatment (control); treated with Z-VAD-FMK (20 μM, 3 h) or EtOH (5%, 2 h) alone; or pretreated with Z-VAD-FMK for 1 h, then co-treated with EtOH for another 2 h. (**E**) Quantification results of cells showing cell shrinkage or membrane blebbing. (**F**) Bright field images of HeLa cells without treatment; treated with Z-VAD-FMK (20 μM, 5 h), EtOH (4.3%, 1 h), or STS (250 nM, 4 h) alone; or pretreated with Z-VAD-FMK for 1 h, then co-treated with EtOH for another 1 h, or co-treated with STS for another 4 h. (**G**) Percentage of HeLa cells with membrane blebbing. (**H**) Percentage of cells with cell shrinkage or membrane blebbing.

Previous studies indicated that cell shrinkage and plasma membrane blebbing occur downstream of caspase activation during apoptosis^35–38^. We investigated whether in our experimental conditions, caspase activity was required for cell shrinkage and membrane blebbing of neuronal cells that recovered to normal morphology after the removal of the cell death stimulus. Neuronal cell lines were pre-treated with the pan caspase inhibitor Z-VAD-FMK for 1 h and then exposed to EtOH for 2 h. As shown in Fig. 8A,B,C,D,E, caspase inhibition had no effect on EtOH induced neurite retraction and cell shrinkage, nor on cell membrane blebbing.

We also studied the effect of caspase inhibition on EtOH and STS treated HeLa cells. Results showed that the caspase inhibitor did not inhibit EtOH induced cell shrinkage and membrane blebbing in HeLa cells (Fig. 8F,G). In STS treated HeLa cells, the caspase inhibitor also did not inhibit cell shrinkage, but did have significant, yet partial, inhibition on STS induced membrane blebbing and formation of structures, resembling apoptotic bodies (Fig. 8F,H).

## Discussion

In the present study, we investigated the reversibility of an activated cell death program in neuronal cells. We explored this by removing cell death stimuli at stages when cells already showed morphological and biochemical hallmarks of apoptosis, prior to the actual death of the cell. We found that cells could recover from many different stages of cellular damage. In all these reversible stages, cytochrome c was still confined to the mitochondria. However, cells that had released mitochondrial cytochrome c into the cytosol, showed no recovery.

Apoptosis is a multistage process of regulated cell death, in which various events associated with loss of viability can occur both at the morphological and molecular level, step by step or simultaneously^1^. Although the precise point of no return may vary in different cell types and conditions, late stages of apoptosis are traditionally considered to be important checkpoints after which cell recovery is no longer possible. These include mitochondrial release of cytochrome c, nuclear fragmentation, and formation of apoptotic bodies^2,39,40^. We studied the reversibility of multiple stages of the cell death program in various neuronal cell lines. In order to demarcate whether the cells had entered late-stage apoptosis, we measured mitochondrial cytochrome c release in fixed or living cells.

Cell shrinkage, membrane blebbing, mitochondrial fragmentation, exposure of phosphatidylserine, and nuclear condensation are all hallmarks of apoptosis^2^. Previous research reported that more than 90% of cells could recover from all these cell death events in multiple cell types treated with diverse cell death stimuli^21,22^. Similarly, we observed a high cell recovery (> 90%) in various neuronal cell lines (Figs. 2, 3, and 4). In our study, we also observed reversible ROS generation and the elevation of intracellular Ca^2+^ (Fig. 2), which are early signs in apoptosis^41^. By studying the distribution of cytochrome c during these reversible cell death events (Figs. 3 and 4), we noticed that in these stages there was no mitochondrial cytochrome c release detectable. Therefore, we conclude that the recovered cells had not entered the late, execution stage of apoptosis. Since there are reports that cell shrinkage and plasma membrane blebbing can occur downstream of caspase activation^35–38^, we examined this with the pan caspase inhibitor Z-VAD-FMK. The results indicated that in our experimental conditions, these events are independent of caspase activation (Fig. 8) and occur during the early, still reversible phase in neuronal cell death.

MOMP, which is part of the intrinsic apoptotic pathway, has been regarded as a point of no return for long time. Mitochondrial outer membrane integrity is highly controlled, primarily through interactions between pro- and anti-apoptotic members of the B cell lymphoma 2 (BCL-2) protein family^5,6^. Following MOMP, cytochrome c is released from mitochondria into the cytosol where it triggers activation of effector caspases^42^. Activated effector caspases cause rapid proteolysis of hundreds of functional and structural substrates resulting in the demise of the cell^43^. Cells can also die after MOMP in the absence of caspase activation through a process termed caspase-independent cell death (CICD)^13^. However, recent studies on anastasis indicated that MOMP is not necessarily a point of no return^22–24,26^. To study whether neurons can recover after MOMP, we generated stably Cyto.c-GFP expressing SH-SY5Y cells and used Cyto.c-GFP release as marker of MOMP. In these cells, MOMP will cause transition from a spotty staining of mitochondria into a diffuse staining of cytosol as visualized with confocal microscopy^32^. By monitoring individual differentiated SH-SY5Y cells before, during, and after exposure to lethal dosage of EtOH, Glutamate, and MPP + with live cell imaging, we clearly observed cytochrome c release from mitochondria into the cytosol. Yet, none of the SH-SY5Y cells that had released cytochrome c could reverse the dying process and recover after removal of the cell death stimulus by washing (Fig. 5). This suggests that MOMP is the point of no return for neuronal cells with an activated cell death process.

To understand why SH-SY5Y cells cannot recover from cytochrome c release, we studied EtOH and STS treated HeLa cells, as it has been reported that these cells could recover from cytochrome c release, caspase activation, PARP activation, cell shrinkage, membrane blebbing, and formation of apoptotic bodies^22–24,26^. On the level of the whole cell population, we observed that both EtOH and STS induced cytochrome c release, caspase and PARP activation in a time dependent manner. At the same time, cell viability quantification indicated that vast majority of cells failed to survive from cytochrome c release and caspase activation (Fig. 6). In order to see whether there were any exceptions of dying HeLa cells that could recover; we monitored individual HeLa cells expressing Cyto.c-GFP before, during, and after EtOH or STS treatment with live cell imaging. Yet, we did not observe any HeLa cells that had released cytochrome c and still could reverse the dying process and recover (Fig. 7). Taken together, our results suggest that recovery after cytochrome c release is not a general phenomenon in EtOH and STS treated HeLa cells.

With respect to recovery from stages before cytochrome C release, we noticed a small difference between EtOH and STS treated HeLa cells. Quantification of the fate of the monitored cells showed that 94% of HeLa cells with mitochondrial fragmentation and without cytochrome c release could recover to normal morphology after the removal of EtOH. In the STS model, only 39.5% of cells with mitochondrial fragmentation and without cytochrome c release recovered after the removal of STS, and 35.5% of cells without mitochondrial fragmentation and cytochrome c release also failed to recover (Fig. 7). An explanation for the reduced recovery in the STS model may be that, in contrast to EtOH, STS binds its substrates with high affinity and washing with fresh medium is not efficient to remove all STS from the cells.

This lack of recovery in our HeLa cells apparently deviates from the results in reports of anastasis, in which individual HeLa cells experienced cytochrome c release and caspase activation and still rapidly recovered to normal morphology within 1–2 h after the removal of EtOH^22,23,26^. Explicit quantification of the percentage of cells that could recover from MOMP and caspase activation appeared to lack in these studies, leaving the frequency and general occurrence of anastasis uncertain. On the level of the whole cell population, these studies used Western blot to detect distribution of cytochrome c, activation of caspase and PARP during and after EtOH or STS treatment in HeLa cells. Results showed that cytochrome c translocated to the cytosol, and cleaved caspase 3 and PARP showed up during exposure to cell death stimuli. After removal of the cell death stimuli, cytochrome c completely disappeared from the cytosol; cleaved caspase 3 and PARP disappeared altogether. Similar results were reported for EtOH or DMSO treated mouse primary liver cells and NIH3T3 cells^22,24^. These Western blot results suggest that virtually all cells that had experienced cytochrome c release and activation of caspase and PARP, had reversed the dying process and recovered. In contrast, we found that there was more cleaved PARP protein expression after removing EtOH and STS compared with the protein expression during EtOH and STS treatment (Supplemental Figure 1). This result is consistent with our cell viability counts, which showed that there was more cell death after removing EtOH and STS compared with cell death during exposure to EtOH and STS (Fig. 6). Clearly then, we could not reproduce key findings of anastasis, i.e. recovery of HeLa cells after cytochrome c release. One possible reason may be the heterogeneity of HeLa cell lines, differing between laboratories^44^.

In previous studies, MOMP was classified as “limited MOMP” or “widespread MOMP^25,45^”. There are reports that cells could recover from incomplete MOMP and sublethal activation of caspase^45–48^. However, all these studies were performed at the level of cell populations. This makes it difficult to distinguish cells that had undergone MOMP and caspase activation from cells that had not, and it remains challenging to verify whether survived cells actually passed through MOMP or caspase activation. In our study, MOMP was indicated by a detectable fluorescent signal of cytochrome c diffused into the cytosol, which we consider to be “widespread MOMP”. Whether neuronal cells or HeLa cells can recover from limited MOMP will be studied later in individual cells by live cell imaging.

The formation of apoptotic bodies is regarded as the last step of apoptosis. These structures are eventually engulfed by phagocytes in a process known as efferocytosis^49^. A previous study reported that in STS treated HeLa cells and EtOH treated human small lung carcinoma H446 cells, cell fragments resulting from the formation of apoptotic bodies could reunite to reconstitute normal cell morphology after removal of the cell death stimulus^23,26^. In our study, we also observed structures resembling apoptotic bodies, particularly in EtOH treated Neuro-2a cells. After removing EtOH and further culturing cells in fresh culture medium, these apoptotic body-like structures coalesce to form cells with apparently normal morphology (Fig. 4C). Importantly, we found that this phenomenon happened before mitochondrial cytochrome c release (Fig. 4F) and could not be inhibited by the pan caspase inhibitor Z-VAD-FMK (Fig. 8), indicating that this phenomenon occurred in the early, reversible phase of the neuronal cell death process. Whether EtOH induced extracellular vesicles in Neuro-2a cells truly are apoptotic bodies is questionable and needs to be investigated further.

In conclusion, we found that cells of various neuronal cell lines can recover from stages in the cell death process before the release of cytochrome c from mitochondria into the cytosol. After cytochrome c release, removal of the cell death triggers clearly is not sufficient to save the cells. This is contrary to recent publications on recovery of these late stages, a phenomenon known as anastasis. Apparently, at least in our hands, recovery after cytochrome c release, or anastasis, is not a spontaneous intrinsic capacity of cells. To save cells after cytochrome c release, other ways, for example specifically blocking the execution pathways after MOMP, may have to be employed.

Rescuing neuronal cells from imminent death, reversing their course towards cell death, can become an important tool in prevention of neurodegenerative diseases. In the present study, we found that early stages in the cell death pathway, before mitochondrial cytochrome c release are reversible by merely removing the cell death stimulus. Apparently, cells possess a repair and recovery program, which takes over when cell death stimuli no longer prevail. Finding these cells and stimulating their recovery holds a promise for preventing or slowing neurodegenerative diseases.

## Materials and methods

### Cell culture and transfection

The human neuroblastoma cell line SH-SY5Y, mouse neuroblastoma cell line Neuro-2a, mouse hippocampal neuronal cell line HT22 and human cervical cancer cells HeLa were purchased from the American Type Culture Collection (ATCC). SH-SY5Y cells were cultured in 50% EMEM (M5650, Sigma-Aldrich) and 50% Ham's F-12 Nutrient Mix (Gibco™ 11765054,) with 10% fetal bovine serum (FBS; F7524, Sigma-Aldrich), and 1% penicillin–streptomycin (Pen/Strep) antibiotic (Gibco™ 15140122). For differentiation, SH-SY5Y cells were plated at a density of 2 × 10^5^ cell/ml and maintained in the differentiation medium containing 1% FBS and 10 μM retinoic acid (RA; R2625, Sigma) for 5 days. Neuro-2a, HT22 and HeLa cells were cultured in EMEM and DMEM (Gibco™ 11960044), respectively, supplemented with 10% FBS and 1% Pen/Strep. Rat pheochromocytoma 12 (PC12) cells were obtained from Leibniz Institute DSMZ (ACC 159) and cultured in RPMI 1640 medium (61870-010, Gibco™) supplemented with 10% horse serum (HS; H1270, Sigma-Aldrich), 5% FBS, and 1% Pen/Strep. To induce differentiation, PC12 cells were dissociated and plated on PDL (P6407, sigma) and laminin (3400-010-02, R&D Systems) coated cell culture plate in low serum conditions (1% horse serum) and with 50 ng/ml of NGF (NGF; N1408, Sigma-Aldrich) for 6–7 days. All cells were cultured at 37 °C in a 5% CO_2_ incubator.

SH-SY5Y and HeLa cells stably expressing cytochrome c-GFP (Cyto.c-GFP) were generated by transfection with pCDN3.1 Cyto.c-GFP plasmid. Briefly, SH-SY5Y or HeLa cells were seeded in a 24-well plate. At cell confluence of 80%, SH-SY5Y cells were transfected with 1 μg DNA using 3 μl NeuroMag Transfection Reagent (NM50200, OZ Biosciences) per well. HeLa cells were transfected with 0.5 μg DNA using 2 μl Lipofectamine™ 2000 Transfection Reagent (11668030, Invitrogen™) per well. After 24 h, cells were digested with trypsin and reseeded in a 50 ml flask at a low density of 1 × 10^4^ cell/ml and selected with 400 μg/ml G418 (4727878001, Sigma-Aldrich) for 2 weeks to select stably transfected Cyto.c-GFP positive cells.

### Cell viability measurement

Cell viability of differentiated SH-SY5Y cells was assessed using the PrestoBlue assay (A13261, Invitrogen™). SH-SY5Y cells were seeded in 96-well plates (20,000 cells/well) and cultured in differentiation medium. After 5 days, cells were exposed to ethanol (Boom), Glutamate (G8415, Sigma-Aldrich), N-Methyl-4-phenylpyridinium Iodide (MPP + ; ab144783, Abcam). After treatments, PrestoBlue was directly added to the wells (1:10) and incubated at 37 °C for 1 h. Following incubation, the change in fluorescence intensity of the PrestoBlue reagent was measured using CLARIOstar® Plus multi-mode microplate reader (BMG LABTECH) with the excitation/emission wavelengths set at 560/590. Cell viability was calculated following normalization to the control group.

Cell viability of HeLa cells was determined by Hoechst33342 (Sigma-Aldrich) and propidium iodide (PI, Sigma-Aldrich) double fluorescent staining as previously described^50^. HeLa cells were seeded at a density of 2 × 10^5^ cell/ml onto 12-well plate. After 24 h, cells were treated with 4.3% (vol/vol) ethanol or staurosporine (STS, 250 nM) for different durations. After treatments, the cells in each group were stained with Hoechst33342 (5 μg/mL) and PI (5 μg/mL) for 15 min in the dark. The stained cells were observed using Olympus IX81 inverted fluorescence microscopy. Hoechst33342 stains DNA of living and dead cells, and thus labels all cells; PI stains dead cells. To assess cell viability, 20 visual fields were randomly selected from each group.

### Live cell imaging

For live cell imaging, we used FEI CorrSight microscopy equipped with wide-field and an Andromeda spinning disk. The FEI MAPS software, in conjunction with Live Acquisition software (LA, FEI) was used to control the microscope and to capture still and time-lapsed images. The microscope was further equipped with an incubation system able to control temperature (Ibidi) and CO_2_ (Digital Pixel, UK) levels. Cells were seeded in 6-channel μ-Slides (Ibidi, 80606). To monitor the cells over time, cell death stimuli were introduced to the cells through perfusion tubes (Ibidi), which were connected to the cell chamber. When cell death stimuli were removed, fresh medium was then introduced to the chamber through these tubes. Fluorescence signals of nuclei, mitochondria, and Cyto.c-GFP were visualized by Andromeda spinning disk. Z-stacks consisted of 10 planes with a Z-interval of 1 μm. Images were shown in the merged z-stacks of 10 planes with the maximum fluorescence intensity. Mock-treated cells were imaged in parallel to ensure that imaging and staining procedures were not cytotoxic. We define mitochondrial fragmentation as the mitochondria in cells appearing as small globes, lacking a tubular and interconnected network as previously reported^51^. Representative images were edited in ImageJ software.

### Western blotting

The proteins were extracted from cells using RIPA lysis buffer (89900, Thermo Scientific™) containing Protease Inhibitor Cocktail (78441, Thermo Scientific™). Proteins were separated by 8%–13% SDS-PAGE gel and subsequently transferred to polyvinylidene fluoride (PVDF) membrane. The PVDF membrane was blocked with blocking buffer (37535, Thermo Scientific™) for 1 h at room temperature. After blocking, membranes were incubated over night at 4 °C with diluted primary antibodies against caspase-3 (14220S, Cell Signaling Technology), cleaved caspase-3 (9664S, Cell Signaling Technology), PARP (9532S, Cell Technology), GAPDH (10R-G109a, Fitzgerald Industries), and Actin (A5441, Sigma-Aldrich). The secondary antibodies used were IRDye 800CW donkey anti-rabbit IgG (H + L) and IRDye 680LT donkey anti-mouse IgG (H + L) (LI-COR Biosciences). The membranes were scanned on an Odyssey imaging system (LI-COR Biosciences).

### Reactive oxygen species (ROS) measurement

The level of ROS was measured using DCFDA dye (ab113851, Abcam) according to the manufacturer’s instructions. In brief, SH-SY5Y cells were seeded on 6-channel μ-Slides (80606, Ibidi) and differentiated for 5 days. After treatment, culture medium was removed and cells were loaded with 20 µM DCFDA diluted in dilution buffer for 30 min at 37 °C. Cells were then washed 3 times with culture medium and imaged under FEI CorrSight microscopy. Fluorescence intensity was quantified with ImageJ.

### Intracellular Ca^2+^ measurement

The level of intracellular Ca^2+^ was measured using Fluo 8-AM (ab142773, Abcam) according to the manufacturer’s instruction. In brief, SH-SY5Y cells were seeded on 6-channel μ-Slide (80606, Ibidi) and differentiated for 5 days. Treated cells were incubated with 4 µM Fluo 8-AM diluted in medium for 30 min at 37 °C. Cells were then washed 3 times with culture medium and imaged under FEI CorrSight microscopy. Fluorescence intensity was quantified with ImageJ.

### Detection of phosphatidylserine (PS) exposure

Redistribution of PS to the outer leaflet of the plasma membrane was visualized by incubating cells with annexin A5-FITC as described in previous study^52^. Cells were incubated with 1 µg/ml annexin A5-FITC (donated from Prof. Reutelingsperger’s lab; Biochemistry department, Maastricht University) in the dark for 15 min at 37 °C, then washed for fresh culture medium. Images were acquired using FEI Corrsight spinning disk; Z-stacks consisted of 10 planes with a Z-interval of 1 µm.

### Immunofluorescence

Cells were fixed in 4% paraformaldehyde (PFA) for 30 min at 4 °C followed by permeabilization in 0.1% Triton X-100 for 10 min at room temperature and blocked with 1% Bovine Serum Albumin (BSA) for 1 h at room temperature. For immunostaining, cells were incubated with primary antibodies against cytochrome c (5 μg/mL; 33–8200, Invitrogen™) and TOM20 (1:50; 42406S, Cell Signaling Technology) overnight at 4 °C. After carefully rinsing in PBS, cells were incubated with a second antibody conjugated with Alexa 488 or 594 (1:500, Invitrogen™) for 1 h at room temperature. The nucleus was labeled by DAPI (D9542, Sigma-Aldrich). The cells were then observed and photographed with FEI CorrSight.

### Statistics

Data were obtained from at least three independent experiments and presented as means ± SEM. Statistical analyses were performed throughout using GraphPad Prism version 9.4.1. For the comparison between more than two groups, values were evaluated by one-way ANOVA followed by a Tukey Kramer test. *p* value less than 0.05 was considered statistically significant.

## Supplementary Information

Supplementary Information.

## Data Availability

All data generated during this study are included in this article (and its supplementary information files).
