# Supplementary material for: Stressed neuronal cells can recover from profound membrane blebbing, nuclear condensation and mitochondrial fragmentation, but not from cytochrome c release
Source: Sci Rep. 2023 Jul 8;13:11045. doi: 10.1038/s41598-023-38210-w (PMC10329692; doi:10.1038/s41598-023-38210-w)

Stressed neuronal cells can recover from profound membrane blebbing, nuclear condensation and mitochondrial fragmentation, but not from cytochrome c release

Wenting You [1,2,3], Tao Zhou [1], Kèvin Knoops [4], Tos T.J.M. Berendschot [1], Marc A.M.J. van Zandvoort [5,7], Wilfred T.V. Germeraad [6], Birke Benedikter [1], Carroll A.B. Webers [1], Chris P.M. Reutelingsperger [2,*] and Theo G.M.F. Gorgels [1,*]

Supplemental Figure 1

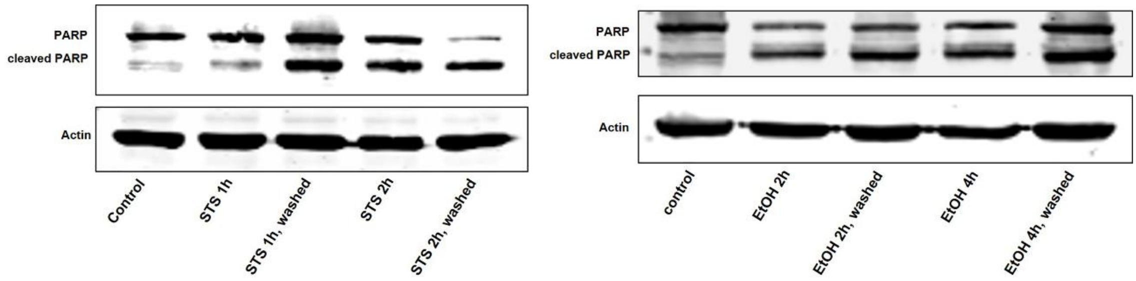

Supplemental Figure 1. Western blot analysis of the protein expression of PARP and cleaved PARP of control, EtOH (4.3%) or STS (250 nM) treated, and washed (15 h) in HeLa cells.

Original full length western blots

Source data_Fig.6C_pro-caspase3

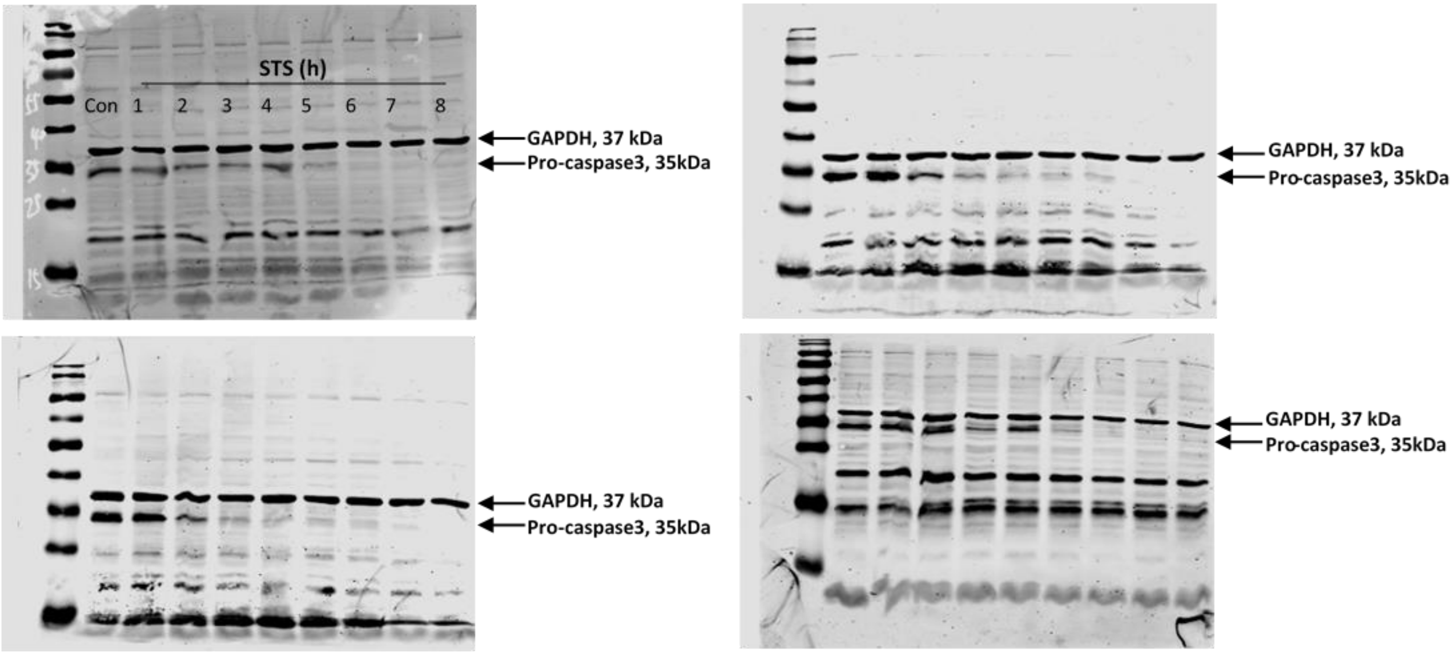

Source data_Fig.6C_cleaved-caspase3

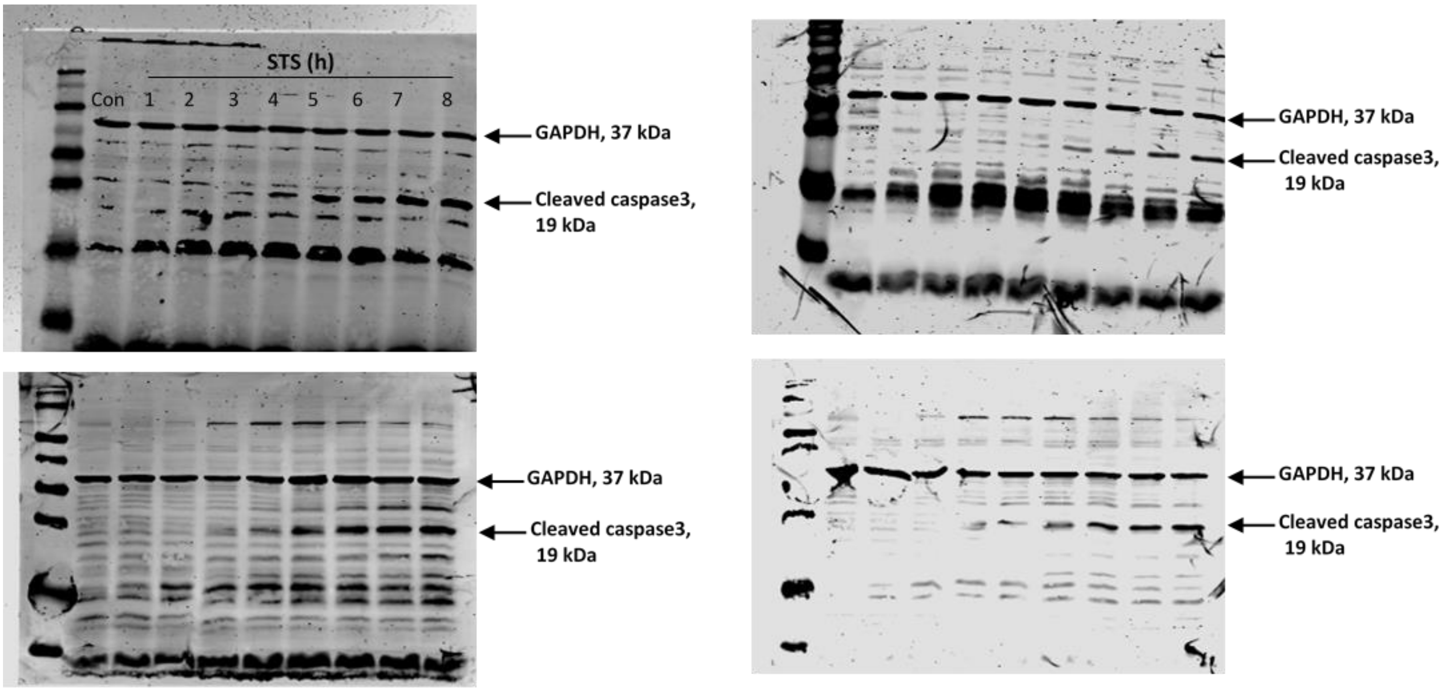

Source data_Fig.6C_PARP and cleaved-PARP

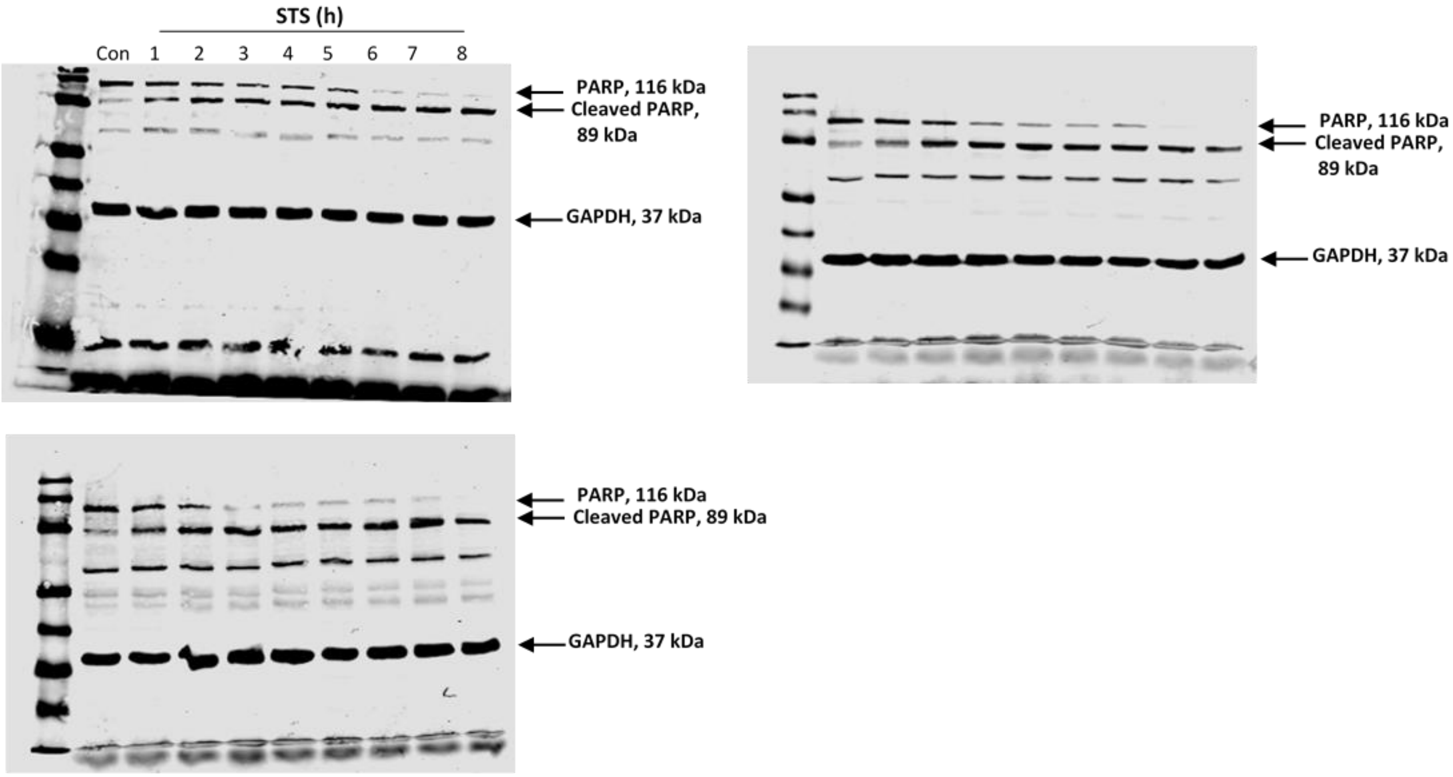

Source data_Fig.6G_pro-caspase3

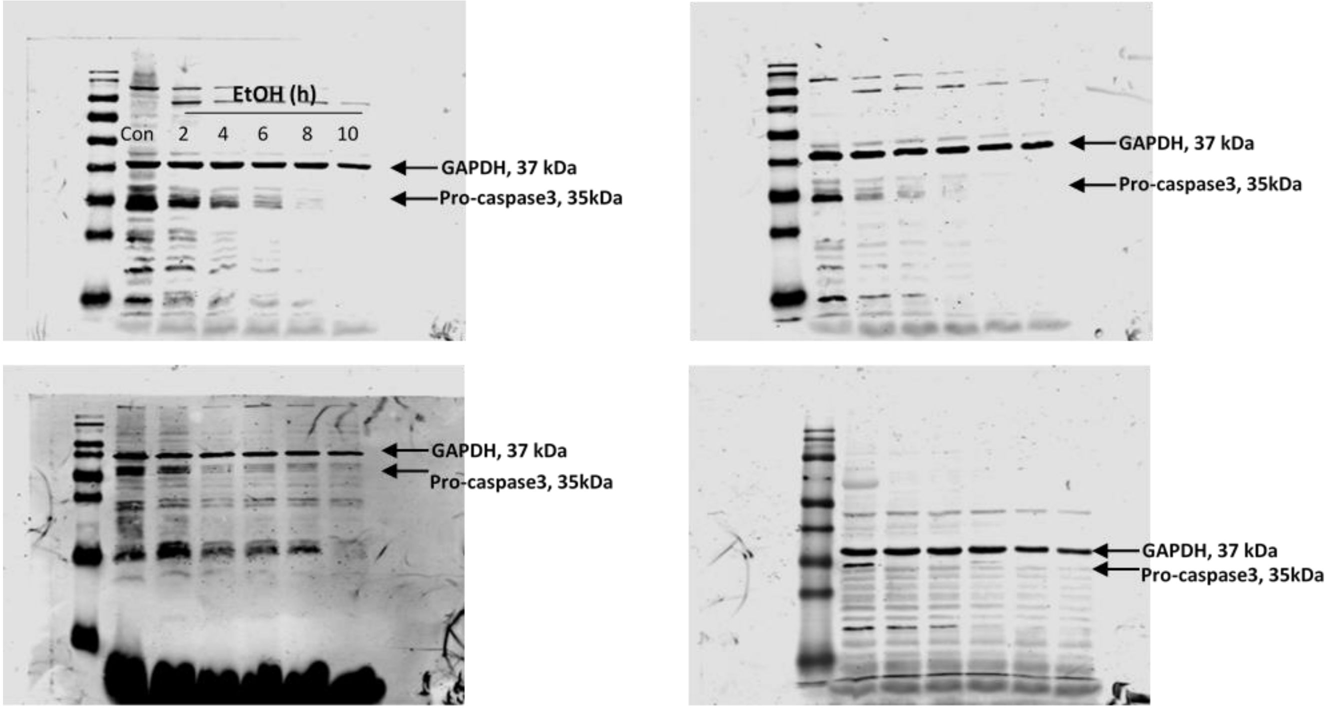

Source data_Fig.6G_cleaved-caspase3

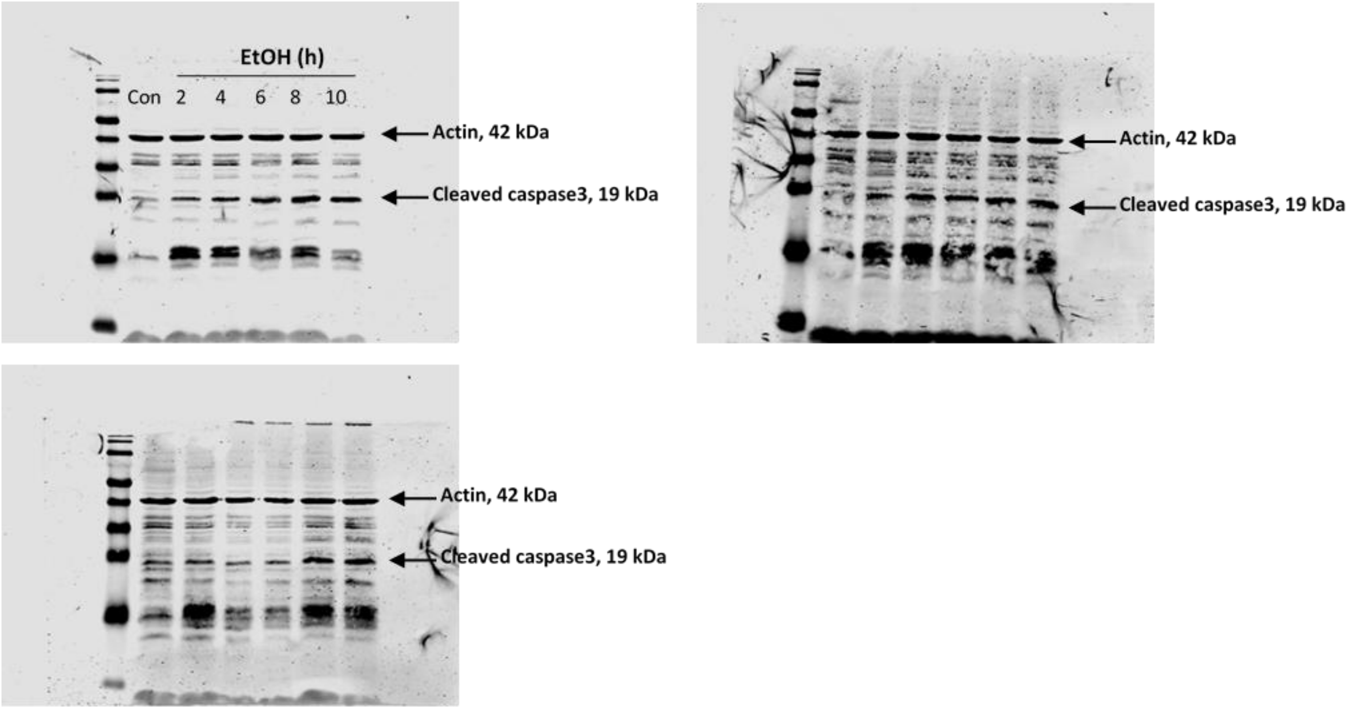

Source data_Fig.6G_PARP and cleaved-PARP

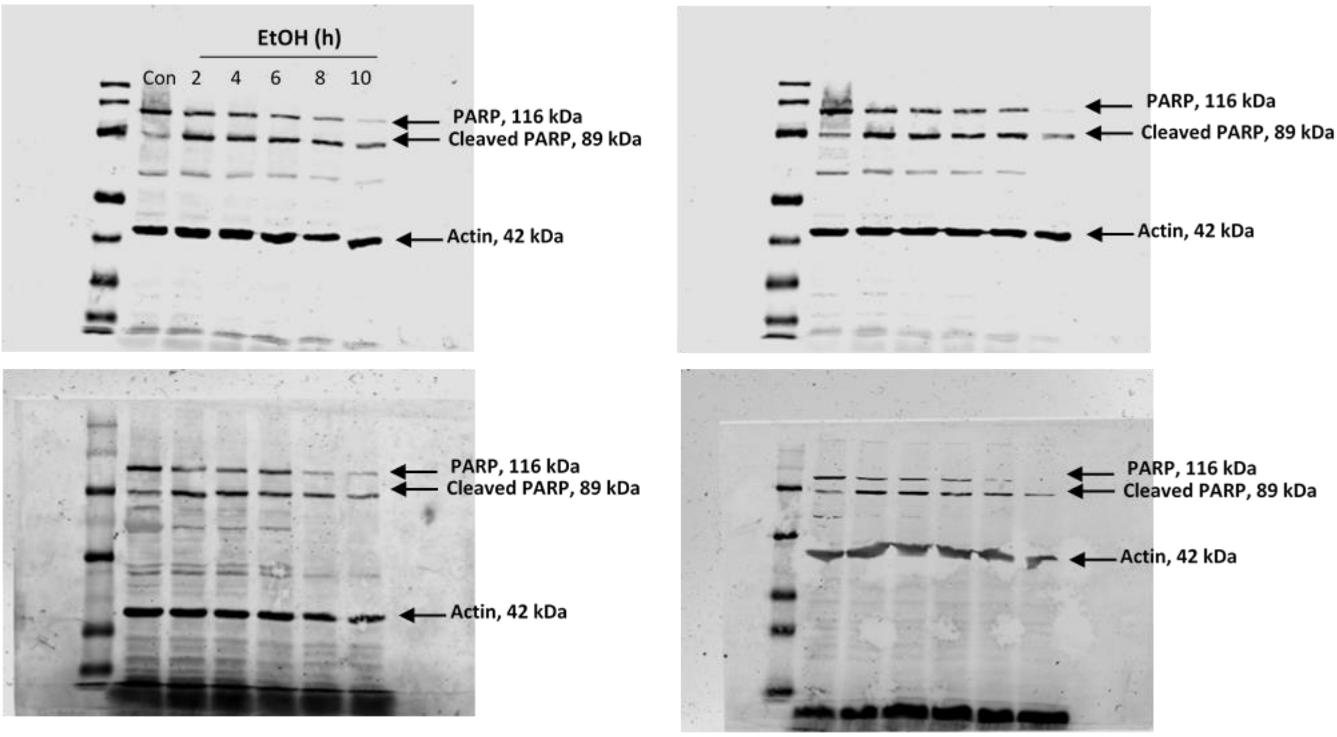

Source data_supplemental Fig.1_PARP_STS model

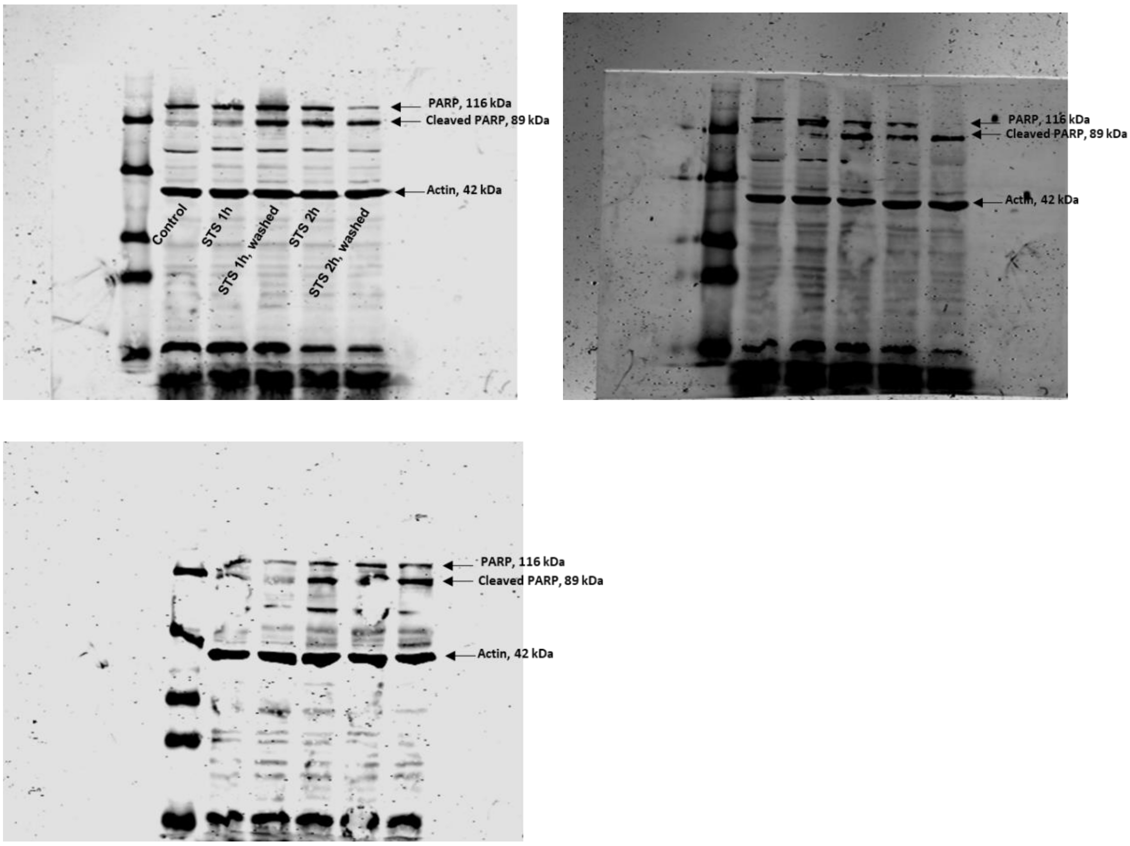

Source data_supplemental Fig.1_PARP_EtOH model

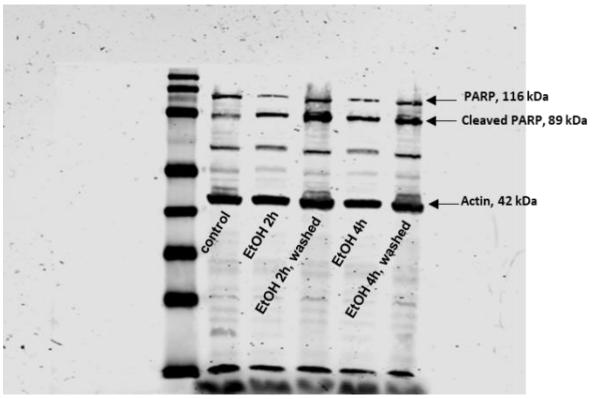

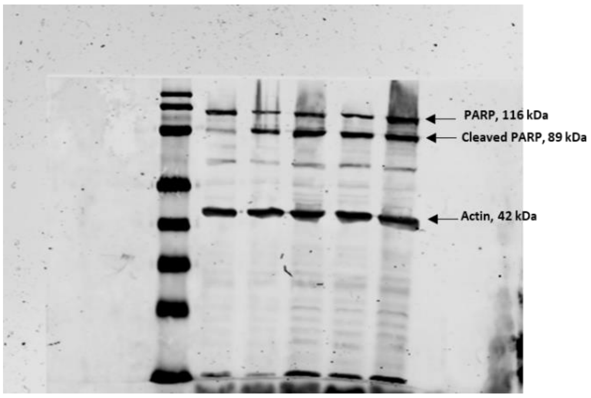

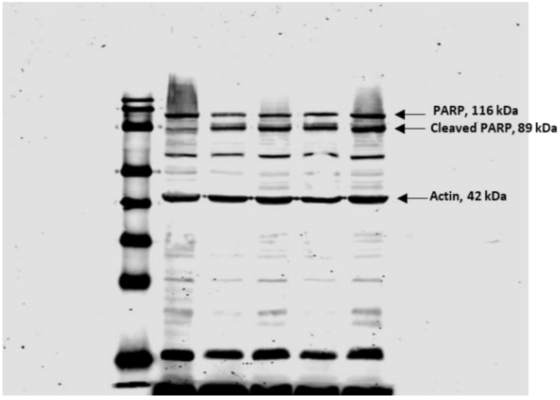

Supplement: Supplementary file 1 — Supplementary Information. [file 41598_2023_38210_MOESM1_ESM.pdf]
